# Cardiocerebral Infarction Presenting in a Neurosurgical Emergency: A Case Report and Literature Review

**DOI:** 10.7759/cureus.65124

**Published:** 2024-07-22

**Authors:** Haruka Kume, Takuma Maeda, Eisuke Tsukagoshi, Takeshi Ogura, Shigehiro Ohmori, Hiroki Kurita

**Affiliations:** 1 Department of Neurosurgery, Kurosawa Hospital, Takasaki, JPN; 2 Department of Neurosurgery, Saitama Medical University International Medical Center, Hidaka, JPN

**Keywords:** 12-lead ecg, thrombolysis, t-pa, cardiocerebral infarction, acute myocardial infarction, acute ischemic stroke

## Abstract

Cardiocerebral infarction (CCI), the simultaneous occurrence of acute ischemic stroke and acute myocardial infarction (AMI), is a rare but critical condition. However, the optimal treatment strategy, particularly regarding the use of tissue plasminogen activator (t-PA), remains unclear. This case report describes a patient with CCI diagnosed during a neurosurgical emergency. A 67-year-old man with a history of hypertension presented with sudden right hemiparesis and sensory aphasia 30 minutes prior to hospital arrival. Diffusion-weighted magnetic resonance imaging revealed acute cerebral infarction in the left middle cerebral artery territory but without large-vessel occlusion. Routine electrocardiography (ECG) showed ST-T elevation in leads V1, V2, II, III, and aVF (augmented vector foot). Subsequent blood tests confirmed positive troponin T and elevated creatine kinase levels. Despite the absence of reported AMI symptoms, the patient received a diagnosis of CCI. Due to the uncertain time of AMI onset and to expedite transfer to the percutaneous coronary intervention (PCI) unit, t-PA administration was withheld. Upon transfer, dual antiplatelet therapy with aspirin (200 mg) and clopidogrel (300 mg) was initiated. Emergency coronary angioplasty successfully treated a 99% stenosis of the left anterior descending artery (#7). The patient’s post-procedure course was uneventful. After 18 days, he was transferred to a rehabilitation hospital with a modified Rankin Scale score of 3. This case highlights the importance of routine 12-lead ECG in neurosurgical emergencies, regardless of presenting symptoms like chest pain. While guidelines support the use of t-PA in CCI, its administration requires careful consideration due to specific risks, including cardiac rupture and limitations on antithrombotic therapy within the first 24 hours.

## Introduction

Cardiocerebral infarction (CCI), a rare but critical condition, presents with both acute ischemic stroke (AIS) and acute myocardial infarction (AMI) occurring either simultaneously or in close succession [1]. According to the American Heart Association, AIS is defined as an episode of acute neurological dysfunction caused by focal cerebral, spinal, or retinal infarction, and AMI is defined as damage to or death of a portion of the heart muscle due to decreased blood flow and oxygen. The association between the two conditions has recently increased; however, the optimal treatment strategies, including thrombolytic therapy, percutaneous coronary intervention (PCI), and mechanical thrombectomy (MT), remain under investigation [2,3]. Notably, the use of tissue plasminogen activator (t-PA) remains a point of debate due to potential complications following AMI [4]. While t-PA is probable as a safe and effective treatment for AIS, patients with AMI who are treated with t-PA may be at risk of cardiac wall rupture and tamponade [5].

Previous reports were mainly from PCI-ready centers where immediate PCI can be performed [3]. In non-PCI facilities, expedited transfer to a PCI facility may be considered along with drug administration [6]. This report describes a case of CCI diagnosed at a non-PCI neurosurgical facility with a literature review.

## Case presentation

**Figure 1:**
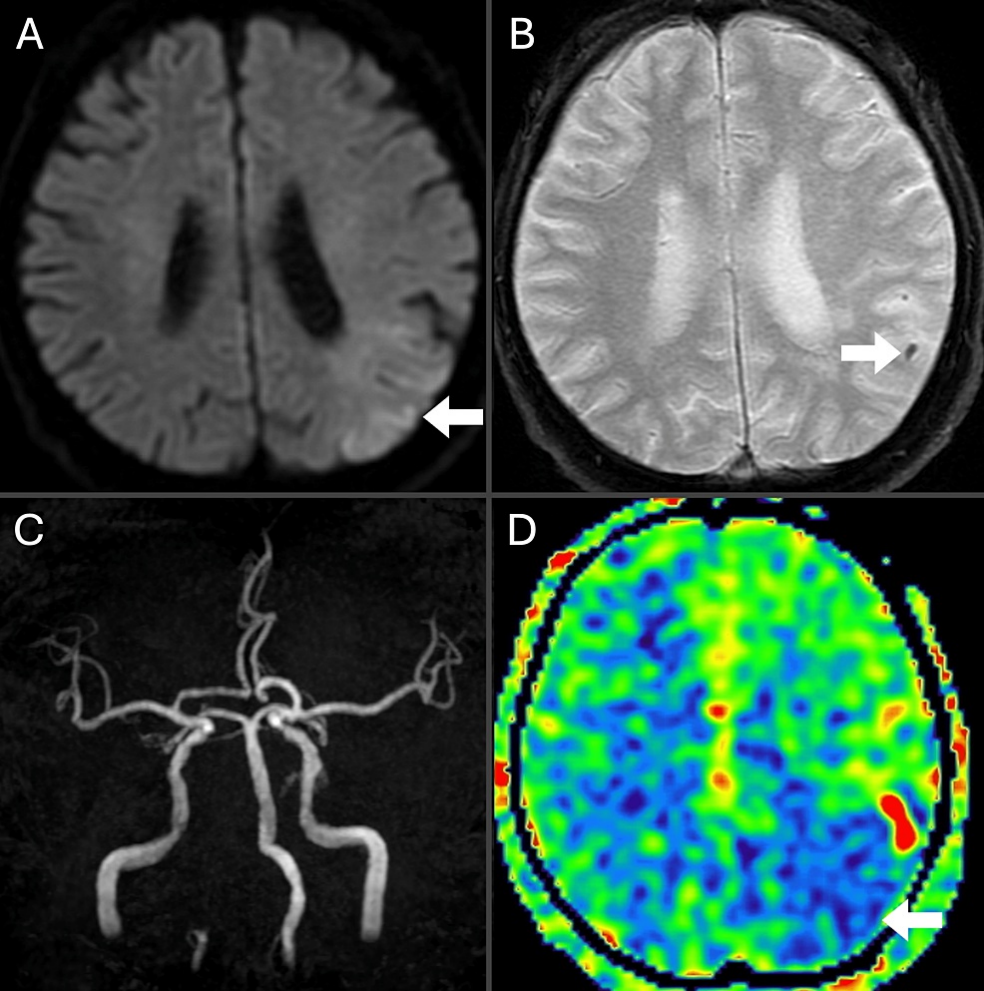
Initial imaging studies. Diffusion-weighted imaging (DWI) showed acute ischemic stroke (AIS) in the left middle cerebral artery (MCA) territory with a DWI-ASPECTS (Alberta Stroke Program Early CT Score) score of 10/11 (A, arrow). T2 star-weighted imaging revealed a susceptibility vessel sign on the left parietal artery (B, arrow). Magnetic resonance angiography did not show any stenosis or occlusion of the main MCA trunk (C). Arterial spin labeling perfusion imaging demonstrated reduced blood flow in the territory of the left MCA (D, arrow).

A 67-year-old man with a history of hypertension presented to our hospital with sudden right hemiparesis that began 30 minutes prior to arrival. On admission, he exhibited an altered mental state with a Glasgow Coma Scale score of E4V2M5. Vital signs were stable with a blood pressure of 130/79 mmHg and a heart rate of 61 beats per minute in sinus rhythm. Neurological examination revealed significant right-sided weakness with facial droop and sensory aphasia. The National Institute of Health Stroke Scale score was 21. Diffusion-weighted imaging (DWI) demonstrated AIS in the left middle cerebral artery (MCA) territory with a DWI-ASPECTS (Alberta Stroke Program Early CT Score) score of 10/11 (Figure 1A). T2 star-weighted imaging revealed a susceptibility vessel sign on the left parietal artery (Figure 1B). Magnetic resonance angiography did not show any stenosis or occlusion of the main MCA trunk (Figure 1C). Arterial spin labeling perfusion imaging further confirmed reduced blood flow in the territory of the left MCA (Figure 1D).

**Figure 2:**
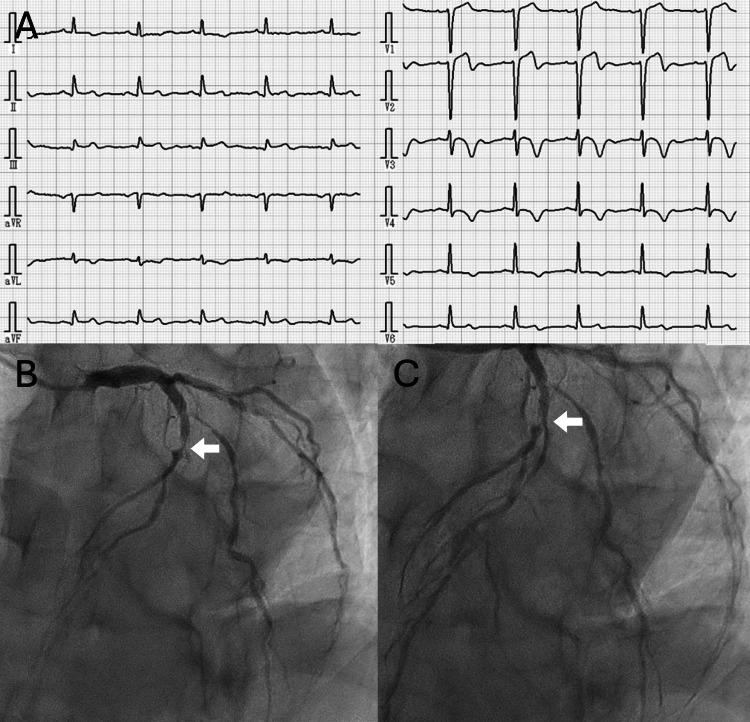
Electrocardiography (ECG) and percutaneous coronary intervention. ECG revealed ST-T wave elevation in leads V1, V2, II, III, and aVF (augmented vector foot), and negative T waves in leads V2–V5 (A). Coronary angiography showed 99% stenosis in the left anterior descending artery (#7) (B, arrow). Emergency plain old balloon angioplasty was successfully performed (C, arrow).

Given the time window for stroke treatment, thrombolytic therapy was initially considered. However, electrocardiography (ECG) revealed ST-T wave elevation in leads V1, V2, II, III, and aVF (augmented vector foot), and negative T waves in leads V2-V5 (Figure 2A), suggestive of AMI. Blood tests confirmed elevated cardiac enzymes (positive troponin T and creatine kinase (CK) levels, 721 U/L). Based on these findings, the diagnosis of CCI was established.

Since the exact timing of the AMI onset was unclear and to facilitate a faster transfer to a PCI facility, thrombolytic therapy for the stroke was withheld. Upon arrival at the PCI center, coronary angiography revealed 99% stenosis in the left anterior descending artery (#7) (Figure 2B). Emergency plain old balloon angioplasty was successfully performed (Figure 2C). Prior to the procedure, dual antiplatelet therapy with aspirin 200 mg and clopidogrel 300 mg, along with anticoagulation with 5000 units of intravenous heparin, was initiated to prevent blood clot formation. The following day, apixaban (5 mg, twice daily) was introduced for long-term prevention of intracardiac thrombosis.

Magnetic resonance imaging on the same day showed no new signs of ischemic stroke or bleeding in the brain. While the patient’s hemiparesis improved, some residual sensory aphasia and cognitive impairment persisted. He did not experience any symptoms related to the AMI, and his blood pressure remained stable. Eighteen days after the initial stroke onset, the patient was transferred to a rehabilitation facility with a modified Rankin Scale score of 3.

## Discussion

This case report describes a patient with CCI diagnosed at a non-PCI neurosurgical facility. He was successfully transferred to a PCI center for treatment of a critical blockage in his coronary artery. Notably, the patient did not receive t-PA due to the co-existing AMI, which allowed for the safe administration of antiplatelet and anticoagulation medications during PCI without complications like cardiac rupture.

CCI, first described in 2010 by Omar et al., is a rare condition involving the simultaneous or sequential occurrence of AMI and AIS [1]. Both AMI and AIS can lead to permanent tissue damage and serious consequences if treatment is delayed. Therefore, rapid diagnosis and intervention are crucial [7,8]. In cases like this, where AMI is diagnosed in a non-PCI hospital, early transfer (within 90-120 minutes) to a facility equipped for PCI is recommended [9,10]. The use of t-PA, a common hyperacute treatment for both conditions, in patients with CCI prior to transfer remains a debated topic due to safety concerns [2].

Table 1 summarizes the data from previously reported CCI cases treated with at least one of t-PA, MT, and PCI [11-22]. The mean patient age was 62.6 years with a mean National Institutes of Health Stroke Scale (NIHSS) score of 16.3. Among the reported cases, 55% (11/20) received t-PA. The average NIHSS score was lower in the t-PA group (13.5) compared to the non-t-PA group (19.8). Favorable outcomes were achieved in a significantly higher proportion of patients who received t-PA (81.8%, 9/11) compared to those who did not (22.2%, 2/9). The reasons for withholding t-PA included cardiac arrest before t-PA administration (case 2), existing anticoagulant therapy (case 4), refractory hypertension (case 5), elapsed treatment window (case 6), potential need for dual antiplatelet agents therapy prior to PCI (case 10), unknown stroke onset time (case 13 and case 15), and low NIHSS score and no cerebral occlusion (case 18).

**Table 1:** Previous cases of cardiocerebral infarction treated with at least one of t-PA, MT, and PCI. ACA, anterior cerebral artery; F, female; ICA, internal carotid artery; LAD, left anterior descending artery; LCx, left circumflex artery; M, male; M1-4, segments of middle cerebral artery; MCA, middle cerebral artery; MT, mechanical thrombectomy; NIHSS, National Institutes of Health Stroke Scale; P1, P1 segment of posterior cerebral artery; PCI, percutaneous coronary intervention; RCA, right coronary artery; t-PA, tissue-type plasminogen activator. A favorable outcome was defined as a modified Rankin Scale (mRS) score of 0-2. An unfavorable outcome was defined as an mRS score of 3-6.

No.	Author & year	Age (years)	Sex	Symptoms	NIHSS	Cerebral lesion	Coronary lesion	t-PA	MT	PCI	Outcome
1	Maciel et al. (2015) [11]	44	M	Chest pain, dysarthria, hemianopia, hemineglect, hemiparesis, homonymous	11	Right MCA	Unknown	Yes	No	No	Favorable
2	Kijpaisalratana et al. (2017) [12]	64	M	Chest pain, hemiparesis	13	Right M1	RCA	No	No	Yes	Unfavorable
3	Kijpaisalratana et al. (2017) [12]	65	M	Dysarthria, hemineglect, hemiparesis	12	Right M1	LCx	Yes	No	Yes	Unknown
4	Yeo et al. (2017) [13]	45	M	Chest pain, hemiplegia, visual neglect	16	Right ICA	LAD	No	Yes	Yes	Unfavorable
5	Yeo et al. (2017) [13]	53	M	Aphasia, hemiplegia	23	Left M1	LAD	No	Yes	Yes	Unfavorable
6	Yeo et al. (2017) [13]	71	F	Hemiplegia, impaired consciousness, vomiting	27	Right P1	LAD	No	Yes	Yes	Unfavorable
7	Yeo et al. (2017) [13]	55	M	Aphasia, hemiplegia	18	Left M1	Unknown	Yes	No	No	Favorable
8	Yeo et al. (2017) [13]	57	M	Aphasia, hemiplegia	16	Left M1	Unknown	Yes	No	No	Favorable
9	Obaid et al. (2019) [14]	41	F	Facial hemiparesis, syncope	6	Left MCA	LAD	Yes	No	Yes	Favorable
10	Sakuta et al. (2019) [15]	55	F	Conjugate eye deviation, hemiparesis, motor aphasia	23	Left M1	RCA	No	Yes	Yes	Unfavorable
11	Nagao et al. (2019) [16]	86	F	Aphasia, hemiplegia	20	Left M2	LCx	Yes	Yes	Yes	Unfavorable
12	Abe et al. (2019) [17]	73	F	Aphasia, hemiplegia	21	Left M1	LAD, LCx (distal)	Yes	Yes	No	Favorable
13	Nardai et al. (2021) [18]	67	F	Conjugate eye deviation, global aphasia, hemiplegia	21	Left M1	LAD	No	Yes	Yes	Favorable
14	Chen et al. (2022) [19]	76	M	Chest pain, hemiparesis, slurred speech	18	Right ICA	LAD	Yes	Yes	Yes	Favorable
15	Nakajima et al. (2022) [20]	86	F	Conjugate eye deviation, global aphasia, hemiplegia	30	Left M1	RCA	No	Yes	Yes	Unfavorable
16	Chong et al. (2022) [21]	51	M	Unknown	6	Left M2	LAD	Yes	No	Yes	Favorable
17	Chong et al. (2022) [21]	45	M	Unknown	6	Left MCA	RCA, LAD	Yes	No	Yes	Favorable
18	Chong et al. (2022) [21]	67	M	Unknown	4	Unknown	LAD	No	No	Yes	Favorable
19	Bao et al. (2022) [22]	84	M	Hemiparesis, slurred speech	14	Bilateral ACA	RCA	Yes	No	Yes	Favorable
20	Present case	67	M	Aphasia, hemiparesis	21	Left M4	LAD	No	No	Yes	Unfavorable

Intravenous t-PA is a well-established treatment for AIS within 4.5 hours after onset, demonstrably improving outcomes [23]. Similarly, t-PA can be effective in reducing mortality and complications associated with ST-elevated myocardial infarction (STEMI) [24]. However, its efficacy is not as high as primary PCI for STEMI, with only about 60% of patients achieving successful reperfusion after t-PA, defined as grade 3 flow according to the Thrombolysis in Myocardial Infarction classification system [25]. Randomized trials comparing facilitated PCI, in which t-PA is used first, have also shown that primary PCI is associated with a lower mortality rate [26,27]. Currently, t-PA within 12 hours of AMI remains an option when timely PCI (within two hours) is not feasible [6].

Current guidelines from the American Heart Association and American Stroke Association consider t-PA followed by PCI as a reasonable approach for CCI (Class IIa, evidence level C) [7,8]. Similarly, Japanese guidelines allow for t-PA use before PCI in eligible AIS patients with coexisting AMI [28]. However, a significant concern exists regarding the potential complications of t-PA in patients with AMI. Cardiac rupture and tamponade are the most serious reported complications. A study by Mannino et al. described a case of fatal cardiac rupture after t-PA administration for AIS in a patient with AMI and summarized similar cases with a higher mortality rate of 64% [5]. Furthermore, anticoagulant and antiplatelet medications, crucial for preventing future thrombotic events, are typically contraindicated within 24 hours of t-PA due to increased bleeding risk [28]. The safety and efficacy of these antithrombotic therapies within 24 hours of t-PA administration are not well known. While aspirin after t-PA does not improve stroke outcomes and may increase intracranial bleeding, dual antiplatelet therapy is essential for coronary artery stenting, creating a management dilemma [13,29,30]. Therefore, t-PA use in CCI necessitates careful consideration with a cardiologist, particularly for patients likely to require coronary stenting or those at high risk of cardiac rupture (elderly, anterior wall septal infarction, and women) [31]. In this case, we opted to withhold t-PA due to three key factors: (1) the uncertain timing of AMI onset, (2) the requirement for dual antiplatelet therapy before PCI, and (3) the need for rapid transfer to a PCI faculty.

Diagnosing CCI can be complex. Patients often experience impaired consciousness and aphasia, making it difficult for them to communicate symptoms like chest pain. Only 23.5% of patients in previously reported cases reported chest or abdominal pain related to AMI (Table 1). This patient, for example, presented with aphasia, hindering communication of any potential AMI symptoms. A routine 12-lead ECG is a valuable tool for suspecting AMI, even in the absence of chest pain [32]. While ECG alone may not be sufficient to diagnose non-STEMI, it remains an important screening tool [33].

## Conclusions

Diagnosis of CCI can be challenging due to the impaired consciousness and aphasia. Neurosurgeons should focus on routine ECGs as well as neurological signs and imaging in all patients, regardless of reported chest pain. While guidelines support the use of t-PA in CCI, its administration requires careful consideration with cardiologists due to specific risks like cardiac rupture and limitations on antithrombotic therapy within the first 24 hours.
